# Editorial: Current and future trends in gestational diabetes diagnosis, care and neonatal outcomes

**DOI:** 10.3389/fendo.2023.1270472

**Published:** 2023-11-30

**Authors:** Giulio Frontino, Elena Succurro, Rosa Corcoy, Francesco Scialabba, Antonella Poloniato, Marina Scavini

**Affiliations:** ^1^ Pediatric Diabetes Unit, Department of Pediatrics, IRCCS San Raffaele Hospital, Milano, Italy; ^2^ Diabetes Research Institute, IRCCS San Raffaele Hospital, Milano, Italy; ^3^ Department of Medical and Surgical Sciences, University Magna Graecia of Catanzaro, Catanzaro, Italy; ^4^ Research Center for the Prevention and Treatment of Metabolic Diseases (CR METDIS), University Magna Graecia of Catanzaro, Catanzaro, Italy; ^5^ Institut de Recerca de l’Hospital de la Santa Creu i Sant Pau, Translational Medicine, Barcelona, Spain; ^6^ Centro de Investigación Biomédica en Red -Bioingeniería, Biomateriales y Nanomedicina (CIBER -BBN), Madrid, Spain; ^7^ Departament de Medicina, Universitat Autònoma de Barcelona, Bellaterra, Barcelona, Spain; ^8^ Neonatology Unit, Department of Pediatrics, IRCCS San Raffaele Hospital, Milano, Italy

**Keywords:** gestational diabetes (GDM), diabetes, newborn, neonatal hypoglycemia, neonatal care, pregnancy

This Research Topic encompasses fourteen curated contributions, each covering different aspects.

Gestational Diabetes Mellitus (GDM), is defined by the World Health Organization as glucose intolerance or hyperglycemia that is first recognized or appears during pregnancy. Over the past two decades, due to lifestyle modifications and a rise in maternal age, the prevalence of GDM has increased significantly, with studies suggesting rates between 1-26%. The ever-rising incidence of GDM varies from 3% to 21.2% in Asia and 0.31% to 18% globally. In the United Arab Emirates (UAE), GDM prevalence fluctuates between 7.9% and 24.9%, with peaks of 37.7%. These variations are due to different diagnostic criteria, timing, and screening methods, with Oral Glucose Tolerance Test (OGTT) screenings typically performed around 24-28 weeks of gestation.

Bashir et al. conducted a study comparing six GDM diagnostic criteria within the Emirati population of the UAE, revealing incidence rates ranging from 8.4% to 21.5%. The most inclusive diagnostic criteria were represented by the National Institute for Health and Clinical Excellence (NICE 2015) and the International Association of Diabetes and Pregnancy Study Groups (IADPSG). On the contrary, the European Association for the Study of Diabetes (EASD 1996) and the New Zealand Society for the Study of Diabetes (NZSSD) criteria were the least inclusive in this population. These findings emphasize the discrepancies among diagnostic criteria for GDM and highlight the need for a universal accurate set of GDM diagnostic criteria to promote consistent incidence estimates and consequent healthcare planning.

Insulin resistance, a significant factor in the development of GDM in late pregnancy, correlates with a reduced presence of butyrate-producing bacteria. A study by Liu et al. explored the composition and evolution of intestinal microbiota from the second to the third trimester in women with GDM, compared to those with normal glucose tolerance during pregnancy. The study revealed noticeably higher levels of *Scardovia* and *Propionibacterium* in the third trimester compared to the second in the control group, a pattern not observed in the GDM group. *Propionibacterium* is reported to improve insulin resistance. Taken together, these findings suggest that variances in gut microbiota may be implicated in GDM pathogenesis.

Failure to manage GDM adequately can lead to short-term and long-term adverse maternal and neonatal outcomes. Short-term maternal complications can encompass preeclampsia, cesarean section, and polyhydramnios, while long-term issues might include post-pregnancy progression to diabetes mellitus, affecting both younger and older women. Furthermore, placental health may also be influenced by maternal age. An elevated risk for placental abruption and placenta previa was identified only in younger women with GDM. However, while the risk for polyhydramnios and preeclampsia was increased in both age groups, an additive interaction of GDM and advanced maternal age was observed (Li et al.). Fetal and neonatal complications can encompass congenital malformation, neonatal death, stillbirth, macrosomia, obstetric trauma, shoulder dystocia, and neonatal hypoglycemia. Early identification and management of women at risk for GDM is crucial, as exposure to intrauterine hyperglycemia before 24–28 weeks of gestation can contribute to abnormal fetal growth and development. The impact of GDM on offspring’s auxological parameter is observable at birth and may persist throughout late childhood. A study by Li et al. reported that GDM that is adequately controlled without the need for drug therapy, was associated with regular birth auxological parameters. However, the same children showed a weight gain during infancy that lagged behind compared to those of the control group.

Maternal anthropometric and metabolic factors, as well as fetal metabolic parameters, exert unique influences on the physical growth parameters of offspring during their inaugural year of life. A research study conducted by Antoniou et al. revealed that certain maternal metabolic parameters, such as high-density lipoprotein cholesterol (HDL), demonstrated a negative correlation with the physical growth measurements of offspring at birth and at 6-8 weeks. On the other hand, glycated hemoglobin (HbA1c) exhibited a positive correlation with offspring growth measurements at the one-year mark. Maternal physical anthropometric parameters, including body mass index (BMI) and fat mass, were predictors of larger physical measurements in offspring at birth. In addition, there was a positive correlation between cord blood HDL and birth weight. In contrast, cord blood insulin, C-peptide, and homeostasis model assessment-insulin resistance (HOMA-IR) demonstrated positive correlations with birth and 6-8 weeks measurements, but they were negatively associated with measurements at one year.

Li et al. created a predictive tool for GDM, employing a variety of fundamental clinical factors easily and inexpensively obtained through patient history and routine blood tests. Factors such as maternal age, blood urea nitrogen (BUN), fibrinogen to albumin ratio (FAR), blood urea nitrogen to albumin ratio (BUN/ALB), and blood urea nitrogen to creatinine ratio (BUN/Cr) were included. This model shows potential for wide application in less developed and developing countries where GDM rates are sharply increasing.

In a separate study, Hu et al. suggested the use of the extreme gradient boosting (XG boost) machine learning algorithm to predict GDM. This model employed twenty predictors incorporating demographic data, clinical attributes, and laboratory parameters. In comparison to the logistic regression (LR) model, which used only four predictors: history of GDM, age, levels of glycated hemoglobin (HbA_1c_), and mean arterial pressure, the XG boost model demonstrated superior accuracy.

Among the glucose parameters for identifying women at risk of GDM, we would like to emphasize HbA1c, which reflects the glucose status of the preceding few weeks. High HbA1c levels and those at the upper normal limit at the time of GDM diagnosis could indicate poor glucose control during early pregnancy. Several studies have reported that adverse outcomes in early pregnancy may be predicted by elevated HbA1c. The study by Muhuza et al. confirmed that patients with HbA1c ≥ 5.5% at the time of diagnosis had a significantly increased risk of macrosomia, preterm delivery, pregnancy-induced hypertension (PIH), and primary cesarean section.

The GDM predictive models mentioned above, developed by Li et al. and Hu et al. could aid the clinical management of pregnant women at early gestational ages and prevent the onset of GDM in at-risk individuals through lifestyle changes, which may not be effective if initiated at later stages.

Close counseling and follow-up during pregnancy in women at risk for GDM lead to better glucose control, reduced HbA1c levels, improved health, and better pregnancy outcomes. Recently in China, the identification and treatment of women with GDM have enabled a reduction in the prevalence of macrosomia and large for gestational age (LGA), probably secondary to superior glucose control (He et al.).

Maternal dyslipidemia is a common occurrence in pregnancy. Particularly, hyperlipidemia is frequently observed in the latter half of pregnancy and is considered a necessary biologically mechanism for the fetus’ energy supply. Insulin resistance and a relative lack of insulin secretion in pregnancies complicated by GDM result in higher serum triglycerides (TRG) and lower HDL-cholesterol. You et al. demonstrated that the triglyceride to high-density lipoprotein cholesterol ratio (TRG/HDL) at 10-14 weeks was positively associated with GDM, and was superior to TRG, HDL, low-density lipoprotein (LDL), and HOMA-IR for predicting GDM.

The management of GDM includes appropriate glucose control, achieved through non-pharmacological and pharmacological therapy. Continuous glucose monitoring provides glucometrics that may be more insightful that regular capillary blood glucose measurements. The study by Dingena et al. showed that there was an increased glucose variability during the day albeit within the normal range. Interestingly, nighttime readings showed prolonged periods of lower glucose levels with relatively less glucose variability.

The primary treatment for GDM typically consists of dietary and lifestyle modifications. A study by Bashir et al. (1) suggested that 80% of women with GDM can achieve normal glucose levels through diet and lifestyle modification alone. Pharmacological therapy for GDM management is required in only 17-30% of cases, but in the study by Brzozowska et al., only 33% of GDM patients achieved an adequate glucose control with diet alone. These authors identified fasting plasma glucose as a predictor of the requirement for pharmacologic treatment, in line with factors identified in previous studies: early GDM diagnosis, a family history of diabetes, non-European ethnicity, advanced age, elevated fasting blood glucose level, HbA_1c_ at GDM diagnosis, and an elevated pre-pregnancy BMI. Continuous glucose monitoring also allow women and healthcare professionals to ascertain whether lifestyle changes are effective in achieving adequate glucose control, or whether pharmacological treatment is necessary.

Although insulin is the current standard treatment, dose titration is often challenging due to risks of glucose variability and hypo-/hyperglycemia. Frequent glucose monitoring and additional education is also a burden. Over the last 20 years, oral hypoglycemic agents (OHAs), primarily glyburide and metformin, have been used. Despite the increasing amount of evidence supporting the use of metformin for GDM (2), the American Diabetes Association (ADA) and American College of Obstetricians and Gynecologists (ACOG) still recommend insulin as the first line of treatment if glucose targets are not achieved with lifestyle interventions. This recommendation stems from the lack of evidence regarding the long-term safety of other drug treatments. On the contrary, the National Institute for Health and Care Excellence (NICE) and Canadian guidelines recommend metformin as the first line treatment. Metformin reduces hepatic gluconeogenesis and increases peripheral glucose uptake without the risk of hypoglycemia.

Several studies support the safety and efficacy of metformin use in GDM as it is associated with less weight gain and a lower risk of neonatal hypoglycemia compared to insulin. Furthermore, metformin’s low cost, low risk of maternal hypoglycemia, and the lack of need for educational programs or intensive glucose control, make it an attractive therapeutic candidate (Tosti et al.).

However, metformin is not universally accepted as the first treatment option due to the lack of consistent evidence of long-term safety, including its diffusion across the placenta, resulting in comparable fetal and maternal drug levels.

Rowan et al. (3) and Paavilainem et al. (4)showed no significant differences in offspring auxological parameters during follow-up. In this study, newborns of metformin-treated mothers with GDM had superior glucose and lipid profiles at follow-up compared to those born from insulin-treated mothers with GDM. On the contrary, the meta-analysis by Tarry-Adkins et al, reported that infants that were exposed to metformin *in utero*, displayed an accelerated post-natal growth (5).

As for technology, although reports have described successful use of insulin pumps in patients with GDM, their use in this context is still sporadic. This is due to lack of clear data suggesting a superior cost-effectiveness than conventional treatments.

Beyond insulin and metformin, taurine could serve as another therapeutic alternative due to its insulin-sensitizing effect. A study conducted by Wang et al. investigated the changes in serum taurine throughout pregnancy and found that these levels were markedly lower during the first trimester in women who subsequently developed GDM. Consequently, taurine may also be used as a diagnostic marker since it has demonstrated the potential to boost insulin sensitivity, stimulate insulin secretion, and diminish inflammation and oxidative stress.

In summary, recent research has not only confirmed the increasing prevalence and potential risks of GDM but also brought forth potential predictors, management strategies, and long-term implications. This information bolsters our understanding of GDM and the multifaceted efforts required to address it effectively. Ongoing research into alternative monitoring and treatment strategies, is crucial to diversify the therapeutic options and ensure optimal care for those affected by GDM.

## Author contributions

GF: Conceptualization, Supervision, Writing – original draft, Writing – review & editing. ES: Supervision, Validation, Writing – review & editing. RC: Supervision, Validation, Writing – review & editing. FS: Writing – original draft, Writing – review & editing. MS: Supervision, Validation, Writing – review & editing. AP: Writing – review & editing, Supervision, Validation.
